# Maripa Virus RNA Load and Antibody Response in Hantavirus Pulmonary Syndrome, French Guiana

**DOI:** 10.3201/eid2409.180223

**Published:** 2018-09

**Authors:** Séverine Matheus, Hatem Kallel, Alexandre Roux, Laetitia Bremand, Bhety Labeau, David Moua, Dominique Rousset, Damien Donato, Vincent Lacoste, Stéphanie Houcke, Claire Mayence, Benoît de Thoisy, Didier Hommel, Anne Lavergne

**Affiliations:** Institut Pasteur de la Guyane, Cayenne, French Guiana (S. Matheus, L. Bremand, B. Labeau, D. Moua, D. Rousset, D. Donato, V. Lacoste, B. de Thoisy, A. Lavergne);; Centre Hospitalier de Cayenne, Cayenne (H. Kallel, A. Roux, S. Houcke, C. Mayence, D. Hommel)

**Keywords:** hantavirus, hantavirus pulmonary syndrome, Maripa virus, French Guiana, antibody response, arboviruses, viruses

## Abstract

We report viral RNA loads and antibody responses in 6 severe human cases of Maripa virus infection (2 favorable outcomes) and monitored both measures during the 6-week course of disease in 1 nonfatal case. Further research is needed to determine prevalence of this virus and its effect on other hantaviruses.

Hantaviruses are members of the genus *Orthohantavirus* (family *Hantaviridae*) and are carried by various rodent species, depending on the strain. Humans can be infected by inhalation of aerosolized viruses excreted in the urine or feces of infected rodents. New World hantaviruses in the Americas cause hantavirus pulmonary syndrome (HPS) in humans, characterized by fever, headache, cough, myalgia, and nausea, evolving rapidly to pulmonary edema (*1**,**2*). This respiratory insufficiency is associated with death in 26%–39% of cases, depending on the New World hantavirus species (*3**,**4*).

Following the identification of Sin Nombre virus (SNV) as the etiologic agent of HPS in the United States in 1993, many other hantaviruses have been identified in the Americas (*3**–**6*). In French Guiana, a laboratory-confirmed case of hantavirus infection was reported in a hospitalized patient in 2008; the complete sequence analysis showed that this was a novel hantavirus closely related to the Rio Mamore species called Maripa virus (*7**,**8*).

We describe antibody responses to Maripa hantavirus infection and viral RNA loads in the 6 laboratory-confirmed human cases in French Guiana, measured at admission to the hospital. We also report how these 2 markers evolved during the course of the disease in the most recent hospitalized case-patient, who had a favorable clinical outcome.

## The Study

Since the time hantavirus diagnostic tools were set up at French Guiana’s Institut Pasteur in 2008, a total of 6 severe human cases of infection by native hantavirus have been reported. All the patients were male; the mean age was 54.6 years (range 38–71 years). The mean time from onset of the disease until admission to the hospital was 4.6 days (range 2–7 days). The clinical outcome was favorable for 2 of the patients; 4 died (Table 1). The clinical and biologic parameters of the first 5 confirmed hantavirus cases were reported previously (*9*). The sixth patient was a 47-year-old man who complained of fever, cough, myalgia, and sweating that had been developing over 6 days. He was admitted to the Andrée Rosemon General Hospital in Cayenne, French Guiana, on August 31, 2017. He experienced respiratory failure, requiring rapid transfer to the intensive care unit for intubation and mechanical ventilation. Thoracic radiography revealed bilateral diffuse alveolar pulmonary infiltrates. The patient remained under mechanical ventilation for 18 days and was discharged from the hospital after 23 days with complete clinical recovery. The clinical symptoms of the patient, and his outdoor activities making the contact with rodents possible, led to suspicion of acute hantavirus infection, which was confirmed by molecular and serologic tests. The complete RNA coding sequence of the S RNA segment (GenBank accession no. MG785209) was also generated and compared with those of the other 5 previous hantavirus cases, showing that it corresponded to a Maripa virus infection (*9*).

**Table 1:** Immune response and viral loads on admission in 6 confirmed hantavirus cases, French Guiana*

Characteristic	Patient 1	Patient 2	Patient 3	Patient 4	Patient 5	Patient 6
Year case reported	2008	2009	2010	2013	2016	2017
Age, y	38	56	49	67	71	47
Days of disease at admission	7	4	2	4	4	7
SNV IgM	Positive	Positive	Positive	Positive	Positive	Positive
IgM sum OD†	1.02	1.70	4.72	0.92	2.23	1.79
SNV IgG	Negative	Negative	Negative	Negative	Positive	Negative
IgG sum OD†	0.05	0.01	0.01	0.01	2.05	0.73
Serum viral RNA load‡	5.8	6.6	6.4	5.9	6.0	6.4
Clinical evolution	Favorable	Death	Death	Death	Death	Favorable

*OD, optical density; SNV, Sin Nombre virus. †Adjusted sum OD values (dilution 1:100, 1:400, 1:1,600, and 1:6,400). ‡Virus copy number was determined as log_10_ copies/mL. Primers and TaqMan probe for quantitative PCR were Maripa_qRT2F 5'-GCAGCTGTGTCTACATTGGAGAA-3', Maripa_qRT2R 5'-CCACCAGATCCGCCAACT-3', and Maripa_Probe2 5'-FAM-AAACTTGCAGAACTCA-MGB-3'.

We tested serum samples from the 6 HPS case-patients that were collected on admission at the intensive care unit and the other 7 sequential serum samples provided from case-patient 6 (6 samples during the hospitalization and 1 after discharge). We performed serologic IgM and IgG tests and assayed them for viral RNA quantification (Tables 1,2). We obtained informed consent from the patients, their representatives, or both at admission and before discharge.

**Table 2:** Monitoring of hantavirus antibodies and viral RNA load in sequential serum samples from patient 6, French Guiana*

Characteristic	Days after symptom onset						
	Day 7	Day 12	Day 15	Day 20	Day 25	Day 30	Day 46
SNV IgM	Positive	Positive	Positive	Positive	Positive	Positive	Negative
IgM sum OD†	1.79	1.56	1.50	1.34	1.01	0.72	0.42
SNV IgG	Negative	Positive	Positive	Positive	Positive	Positive	Positive
IgG sum OD†	0.73	1.83	2.20	3.08	4.21	4.71	4.40
Serum viral RNA load‡	6.4	5.4	4.7	4.1	4.0	4.1	0

*OD, optical density; SNV, Sin Nombre virus. †Adjusted sum OD values (dilution 1:100, 1:400, 1:1,600, and 1:6,400).  ‡Virus copy number was determined as logs_10_ copies/mL. real-time PCR Primers and TaqMan probe for quantitative PCR were Maripa_qRT2F 5'-GCAGCTGTGTCTACATTGGAGAA-3', Maripa_qRT2R 5'-CCACCAGATCCGCCAACT-3', and Maripa_Probe2 5'-FAM-AAACTTGCAGAACTCA-MGB-3'.

We assayed all serum samples by IgM capture and IgG ELISA using the protocol described by Ksiazek et al. (*10*). We tested samples against SNV antigen and control antigen using 4-fold dilutions, from 1:100 to 1:6,400. Because of antibody cross-reactivities, positive ELISA findings with SNV antigens indicated infections with New World hantaviruses. The positive criteria were similar to those described by MacNeil et al. (*11*).

The serologic investigations showed that all samples collected at admission had detectable amounts of hantavirus IgM: minimum IgM titers >400 for patients 1, 2, 4, 5, and 6 and a maximum titer of >16,00 for patient 3 (Table 1). These data were similar to those reported in previous work (*11**,**12*). Only patient 5, who died 24 hours after admission, had serum samples positive for hantavirus IgG (titer >6,400). Although the time from the onset of disease and sample collection at admission was different for each of the 6 patients, this single positive hantavirus IgG case may be explained in part by the longer viral incubation period, resulting in the induction of IgG before the appearance of symptoms. A previous study reported that the presence of hantavirus IgG during the first week of infection might be a predictor of survival, but we found no evidence supporting this view (*11*).

To determine the viral RNA load in each serum sample, we performed real-time PCR. Each reaction was performed in duplicate. For absolute quantification, we calculated the exact number of copies of the gene of interest using a standard curve established with plasmid DNA at dilutions from 5 to 5 × 10^7^ copies/mL. The viral RNA loads in the samples collected on admission were 5.8–6.6 log_10_ copies/mL (mean 6.2 ± 0.3 log_10_ copies/mL) (Table 1). These values were similar to those observed in patients infected by other hantaviruses, including patients with mild or moderate symptoms (*13**–**15*)*.* We also observed that the viral RNA load in the 4 fatal cases was 6.2 log_10_ copies/mL, whereas in the 2 nonfatal cases it was 6.1 log_10_ copies/mL. A correlation between hantavirus RNA loads in the serum during the acute phase of disease and the clinical outcome has been hypothesized (*14**,**15*); however, although our study includes only a small number of cases and only severe cases, it provides no evidence supporting this possibility. Presumably, the fatal or nonfatal outcome depends not only on the hantavirus viral load but also on other pathogenic or host factors.

The progression of these antibody responses and viral RNA loads was also followed during the course of disease for patient 6, from admission to the hospital (day 7) until day 46 after the onset of disease (Table 2). IgM titers were high at admission but decreased to become undetectable by day 46. Conversely, seroconversion (IgM to IgG) was observed between day 7 and day 12; these hantavirus IgG titers then increased to 4.4 by day 46. Likewise, viral RNA load evaluated in these 7 sequential serum samples showed a high value at admission (6.4 log_10_ copies/mL), declining by 7 days later to 4.7 log_10_ copies/mL (Table 2). Viral load then remained around 4 log_10_ copies/mL in samples collected on days 20, 25, and 30 and was undetectable on day 46.

## Conclusions

Although limited in sample size, this study found similar results for viral load and immune response in the first 6 cases of Maripa virus infection reported in French Guiana after laboratory-based surveillance began in 2008. Further work is needed to determine the overall prevalence of this hantavirus in French Guiana and also the possible undetected mild or moderate cases induced by Maripa virus infection as reported for other New World hantaviruses (*13**–**15*). Moreover, it would be informative to determine the infectious potential of the virus in the sequential samples to provide a better understanding of the pathophysiology of this infection. Investigations of the immune response to hantavirus, consequences of different viral loads, and the pathologic characteristics of different hantavirus strains would help identify the determinants of disease outcome.

## References

[1] Jonsson CB, Figueiredo LT, Vapalahti O. A global perspective on hantavirus ecology, epidemiology, and disease. Clin Microbiol Rev. 2010;23:412–41. 10.1128/CMR.00062-09 20375360 PMC2863364

[2] Enria DA, Briggiler AM, Pini N, Levis S. Clinical manifestations of New World hantaviruses. Curr Top Microbiol Immunol. 2001;256:117–34. 10.1007/978-3-642-56753-7_7 11217400

[3] Martinez VP, Bellomo CM, Cacace ML, Suarez P, Bogni L, Padula PJ. Hantavirus pulmonary syndrome in Argentina, 1995-2008. Emerg Infect Dis. 2010;16:1853–60. 10.3201/eid1612.091170 21122213 PMC3294556

[4] MacNeil A, Ksiazek TG, Rollin PE. Hantavirus pulmonary syndrome, United States, 1993-2009. Emerg Infect Dis. 2011;17:1195–201. 10.3201/eid1707.101306 21762572 PMC3321561

[5] Montoya-Ruiz C, Diaz FJ, Rodas JD. Recent evidence of hantavirus circulation in the American tropic. Viruses. 2014;6:1274–93. 10.3390/v6031274 24638203 PMC3970150

[6] Centers for Disease Control and Prevention. International HPS cases. 2012 [cited 2017 Apr 1]. https://www.cdc.gov/hantavirus/surveillance/international.html

[7] Matheus S, Djossou F, Moua D, Bourbigot AM, Hommel D, Lacoste V, et al. Hantavirus pulmonary syndrome, French Guiana. Emerg Infect Dis. 2010;16:739–41. 10.3201/eid1604.090831 20350412 PMC3321943

[8] Matheus S, Lavergne A, de Thoisy B, Dussart P, Lacoste V. Complete genome sequence of a novel hantavirus variant of Rio Mamoré virus, Maripa virus, from French Guiana. J Virol. 2012;86:5399. 10.1128/JVI.00337-12 22492924 PMC3347398

[9] Matheus S, Kallel H, Mayence C, Bremand L, Houcke S, Rousset D, et al. Hantavirus pulmonary syndrome caused by Maripa virus in French Guiana, 2008–2016. Emerg Infect Dis. 2017;23:1722–5. 10.3201/eid2310.170842 28930019 PMC5621545

[10] Ksiazek TG, Peters CJ, Rollin PE, Zaki S, Nichol S, Spiropoulou C, et al. Identification of a new North American hantavirus that causes acute pulmonary insufficiency. Am J Trop Med Hyg. 1995;52:117–23. 10.4269/ajtmh.1995.52.117 7872437

[11] MacNeil A, Comer JA, Ksiazek TG, Rollin PE. Sin Nombre virus-specific immunoglobulin M and G kinetics in hantavirus pulmonary syndrome and the role played by serologic responses in predicting disease outcome. J Infect Dis. 2010;202:242–6. 10.1086/653482 20521946

[12] Bostik P, Winter J, Ksiazek TG, Rollin PE, Villinger F, Zaki SR, et al. Sin nombre virus (SNV) Ig isotype antibody response during acute and convalescent phases of hantavirus pulmonary syndrome. Emerg Infect Dis. 2000;6:184–7. 10.3201/eid0602.000213 10756154 PMC2640842

[13] Bellomo CM, Pires-Marczeski FC, Padula PJ. Viral load of patients with hantavirus pulmonary syndrome in Argentina. J Med Virol. 2015;87:1823–30. 10.1002/jmv.24260 26087934

[14] Terajima M, Hendershot JD III, Kariwa H, Koster FT, Hjelle B, Goade D, et al. High levels of viremia in patients with the Hantavirus pulmonary syndrome. J Infect Dis. 1999;180:2030–4. 10.1086/315153 10558964

[15] Xiao R, Yang S, Koster F, Ye C, Stidley C, Hjelle B. Sin Nombre viral RNA load in patients with hantavirus cardiopulmonary syndrome. J Infect Dis. 2006;194:1403–9. 10.1086/508494 17054070

